# A32 PHAGE TREATMENT DELAYS ONSET OF CROHN’S-ASSOCIATED E. COLI DRIVEN COLITIS IN MICE COLONIZED WITH A DEFINED MICROBIOTA

**DOI:** 10.1093/jcag/gwac036.032

**Published:** 2023-03-07

**Authors:** K Jackson, H Galipeau, A Hann, B Coombes, Z Hosseinidoust, E Verdu

**Affiliations:** 1 Chemical Engineering; 2 Medicine; 3 Biochemistry and Biomedical Sciences, McMaster University, Hamilton, Canada

## Abstract

**Background:**

Opportunistic pathogens have been postulated to drive dysregulated inflammation in inflammatory bowel disease (IBD). Indeed, adherent-invasive *Escherichia coli* (AIEC) isolated from IBD patients have pathobiont and pro-inflammatory characteristics. Current treatments for IBD suppress the immune response and do not target key microbial drivers, therefore novel strategies are required.

**Purpose:**

Our aim was to determine whether bacteriophage therapy targeted against AIEC could reduce the severity of *E. coli*-driven colitis in gnotobiotic mice.

**Method:**

Adult germ-free C57BL/6 mice were colonized with altered Schaedler-like flora (ASF) and *E. coli* NRG857c, a Crohn’s disease-associated bacterial isolate. Three weeks later, mice were treated with daily phage (selected by killing curves bioassays against *E. coli* NRG857c) or PBS for 2 weeks (n=6/group). Mice were then exposed to low-dose dextran sulfate sodium (2%; DSS) in drinking water for 5 days, followed by 2 days of water. PBS-treated mice (n=6) that received no DSS were used as additional negative controls. Mice were monitored daily for weight, stool consistency, and occult blood. At sacrifice, colon tissue was collected for histological analysis and fecal contents were cultured to determine bacterial load. In separate experiments, C57BL/6NTac-*Il10*^*em8Tac*^ (IL-10^-/-^) mice were colonized with ASF-like microbiota and *E. coli* NRG857c. Three weeks later, mice (n=5) were treated with weekly phage or PBS (n=5) for 7 weeks. Mice were monitored weekly as described above.

**Result(s):**

Daily phage treatment reduced the severity of clinical symptoms induced by acute DSS administration (p < 0.001 vs. DSS-PBS treated mice). At endpoint, phage treatment was associated with lower histological scores as compared with DSS-PBS controls (p < 0.0001). A 1-log reduction in AIEC bacterial load was observed in phage treated mice as compared with DSS-PBS controls (p < 0.001). In IL-10^-/-^ mice, weekly phage treatment delayed the spontaneous onset of colitis (p < 0.0001 vs. PBS-treated mice). At endpoint, mice treated with phage had lower colitis scores. Reduced weekly AIEC bacterial load was observed in phage-treated mice.

**Conclusion(s):**

Lytic phages, targeting a known AIEC pathobiont isolated from Crohn’s disease patients, ameliorate acute intestinal injury and delay onset of spontaneous colitis. Future work will investigate the mechanisms by which phage therapy prevents and treats colitis, to better inform clinical trial design.

**Disclosure of Interest:**

None Declared

